# The Outcome of the Reconstructive Procedure Using Buccal Pad of Fat Flap and Deep Plane Facelift after Permanent Filler Removal

**DOI:** 10.1007/s00266-026-05836-w

**Published:** 2026-04-20

**Authors:** Samir Ghoraba, Maram Ismail

**Affiliations:** 1 Plastic and Reconstructive Surgery Department, Tanta University, The Corner Mall, 2nd Floor, New Cairo, Cairo, 31111 Egypt; 2 Plastic and Reconstructive Surgery Department, Cairo University, Cairo, Egypt

**Keywords:** Buccal fat, Facelift, Permanent filler, Deep plane, BFP, Contour irregularities

## Abstract

**Background:**

Deep plane facelift in patients with prior permanent filler injection is a formidable reconstructive and aesthetic procedure. Various reconstructive techniques have been previously described in the literature to address the defects after permanent fillers removal. This study investigates the effectiveness and complication rate of reconstruction of these soft tissue defects using the buccal pad of fat flap.

**Materials and Methods:**

This prospective cohort study was conducted at Opal Aesthetic Center in Cairo from 2016 to 2022. Patients underwent removal of the permanent fillers, followed by a deep plane facelift and reconstruction of the resulting defect using a buccal pad of fat flap. Follow up was done for a minimum period of six months to a maximum of five years to assess contour and complications.

**Results:**

A study of 151 patients, comprising 92.7% females with a mean age of 47.8 years, examined the outcomes of reconstruction using buccal pad of fat flap following permanent filler removal in conjunction with a deep plane facelift procedure. Patient satisfaction was high, with 80.7% rating their outcomes as “very good” or “excellent”. Primary surgeries had an 88.5% satisfaction rate, whereas revision surgeries had only a 33.4% satisfaction rate. Contour irregularities were reported in 10.6% of cases and were linked to the occurrence of complications (*p* < 0.001).

**Conclusion:**

The buccal fat pad flap combined with a deep plane facelift after permanent filler removal is a safe and effective reconstructive method with high patient satisfaction. Primary surgeries generally have better outcomes than secondary revisions. The outcome of the buccal fat pad procedure has shown to be negatively affected by smoking, previous surgeries and the presence of post-operative complications. Future research should refine techniques for secondary cases and investigate treatments to improve tissue quality.

**Level of Evidence IV:**

This journal requires that authors assign a level of evidence to each article. For a full description of these Evidence-Based Medicine ratings, please refer to the Table of Contents or the online Instructions to Authors www.springer.com/00266.

**Supplementary Information:**

The online version contains supplementary material available at 10.1007/s00266-026-05836-w.

## Introduction

The popularity of filler injections has increased significantly in the recent years, with approximately 3.4 million soft-tissue fillers administered in 2020 alone [1]. Although hyaluronic acid (HA) fillers are currently the most widely used injectable fillers worldwide [2, 3], permanent fillers have previously been widely used to correct aging-related wrinkles and restore tissue volume [4, 5]. However, patients usually encounter several delayed and lifelong side effects after permanent filler injection. Complications may manifest as severe issues, including chronic inflammation, late allergic reactions, bacterial infections, injection-induced necrosis caused by vascular occlusion or trauma, development of foreign body granulomas, and the consequences of filler migration [6–12].

Complications from permanent fillers are usually addressed through surgical removal, which involves excising the filler and performing restorative surgery to correct resulting deficits or asymmetries [13, 14]. The process of filler removal can be complex and may require multiple procedures, which can negatively impact the aesthetic results of restorative surgery [15–17]. Consequently, the decision to remove permanent fillers is typically based on the severity of complications and the patient's overall condition [18].

After permanent filler removal, several reconstructive techniques can be utilized to address the resulting soft tissue defects [19]. One such technique is the pedicled buccal fat pad flap. This surgical method involves harvesting autologous buccal pad of fat in the form of an advancement-transposition flap while preserving its blood supply through a pedicle, which helps restore tissue defects at the recipient site [20]. The BFP (buccal fat pad) is a mass of adipose tissue located within the buccal and masticator spaces. It consists of a main body and four extensions: the buccal, pterygoid, pterygopalatine, and temporal. It fills the deep tissue spaces of the midface, confined by the buccinator muscle medially, and by the muscles of facial expression and the deep cervical fascia anterolaterally. It acts as a gliding plane for muscles and as a protective layer for neurovascular structures [11, 21–23]. The advantage of the pedicled flap is that it is less technically demanding, thereby avoiding the need for microsurgical techniques, which leads to shorter operative time and less postoperative care [24].

Despite a consistent increase in publications discussing various reconstructive techniques after permanent filler removal, the literature has not yet demonstrated the superiority of one technique over another, with more studies focusing primarily on free fat transfer [14, 25–27]. Therefore, we conducted this study to provide a facial reconstructive modality following permanent filler removal by evaluating the effectiveness and postoperative complications of the pedicled buccal pad of fat flap.

## Materials and Methods

### Study Design and Setting

This prospective case-series cohort study was conducted at Opal Aesthetic Center in Cairo, between 2016 and 2022.

### Eligibility Criteria

All candidates underwent removal of permanent fillers in conjunction with a deep-plane facelift. The primary indications for surgery were the presence of nodules or indurations, significant asymmetry, severe incapacitating deformities or disfiguring facial edema. Patients’ assignment to the study was based on the need for a reconstructive procedure to address a resulting soft tissue defect. Therefore, the procedure was complemented by reconstructing the defects using a pedicled buccal pad of fat flap.

Patients were excluded from surgery if they had a body mass index (BMI) over 30, experienced a recent infection within three months before the procedure, or had persistent severe malar edema.

### Data Collection

The data were collected from 2016 to 2022. To ensure a comprehensive dataset, demographic information for each participant, including age, gender, and BMI, was gathered alongside other factors such as smoking status, history of previous infections, and whether the procedure was a primary or secondary corrective surgery. Additionally, complications related to the surgical technique were recorded.

### Surgical Technique

A classical facelift incision was utilized, with minimal undermining of the skin to access the deep plane. The incision to enter the deep plane began at the junction of the fixed and mobile superficial musculoaponeurotic system (SMAS), roughly 2 cm in front of the ear lobule, following a line from the lateral canthus to the mandibular angle. Entry into the deep plane was achieved through sharp dissection, followed by careful blunt dissection through the facial spaces to safeguard the branches of the facial nerve. The dissection commenced in the masseteric space, progressed to the buccal space, and concluded in the prezygomatic space, which successfully exposed the facial ligaments. The masseteric, zygomatic, and cervical retaining ligaments were meticulously released to protect the nearby facial nerve branches.

If any permanent fillers beneath the SMAS were found to be in a fluid state, they were removed, while solid permanent fillers were sculpted with caution. In the subcutaneous plane, similar management was applied to permanent fillers through targeted pockets, avoiding extensive dissection to address these fillers. Tissues were irrigated with a generous volume of normal saline combined with 10% povidone-iodine and 10% hydrogen peroxide in a 100:10:5 ratio.

After an initial vectoring of the composite myocutaneous flap, the facial contour was evaluated. Any defects created by removal of the permanent fillers were reconstructed using a pedicled buccal fat pad flap, which was carefully delivered from the sub-SMAS masticatory space after creating a small incision in its capsule. The flap was bluntly dissected and gently mobilized to fill these voids as shown in Video 1. The flap was anchored in place with 4-0 Vicryl® sutures.

The composite myocutaneous flap was then secured to the mastoid fascia posterior to the auricle and to the stable SMAS anterior to the auricle using both 0 Vicryl® and 2-0 Vicryl® sutures respectively, after vertical vectoring. A suction drain was routinely placed in the subcutaneous layer, and no compression dressing was utilized. Typically, the drain was removed after approximately 48 hours, while the bolster dressing was removed after one week. Patients were advised to refrain from sleeping on their sides for two weeks.

## Objectives

### Primary Outcome

Contour correction and symmetry were assessed using a satisfaction scale, based on the Subject's Global Aesthetic Improvement Scale (SGAIS) at the 6^th^ month post-treatment. Patients were self-evaluated for the results to be either described as no improvement (poor), slight improvement (fair), (very good), or marked (excellent) improvement [28].

### Secondary Outcomes

The study focused on assessing complications arising from the facial reconstructive procedures using the buccal pad of fat flap. Complications were defined as any symptoms or signs attributable to this procedure, including seroma, infection, and abscess formation. Contour irregularities were reported by a blinded surgeon who was not involved in patient care and was not provided with any information regarding the surgical technique or procedural details. The minimum follow-up period was six months after surgery, with a maximum of 5 years.

### Statistical Analysis

The data were revised, cleaned, and extracted into an Excel sheet. Statistical analysis was conducted using SPSS (Statistical Package for the Social Sciences) software, version 26. Descriptive statistics were used to present the data. Categorical variables were reported as frequencies and valid percentages. In contrast, numerical variables were expressed as mean ± standard deviation (SD) for normally distributed data and as median with interquartile range (IQR), minimum, and maximum for non-normally distributed data. The normality of the data was assessed using the Shapiro-Wilk test. Fisher's exact test was used to compare two categorical variables. Statistical significance was considered when the P value was less than 0.05.

### Results

This study involved 151 patients who underwent reconstructive surgery utilizing a buccal fat pad flap along with a deep plane facelift following the removal of permanent fillers. The cohort was predominantly female, comprising 92.7%, with a mean (SD) age of 47.8 years. The majority of participants were non-smokers (79.5%) and had a body mass index (BMI) range of 25–30 kg/m^2^ (92.1%). Most of the procedures performed were primary surgeries (86.1%), while a smaller percentage were revisions (13.9%). The primary indications for surgery were the presence of nodules or indurations (37.1%) and significant asymmetry (32.4%), followed by Severe incapacitating deformities (21.2%) and disfiguring facial edema (9.3%), as detailed in Table 1.

**Table 1:** Baseline demographic and clinical characteristics of the patients (*N* = 151)

Factors	Category	Number	Frequency
Gender	Female	140	92.7
	Male	11	7.3
Age group (Years)	Less than or equal to 45	73	48.3
	More than 45	78	51.7
	Mean (SD)	47.78 (8.363)	
	Median (IQR)	46 (10)	
	Min-Max	28-79	
Smoker	Yes	31	20.5
	No	120	79.5
BMI	25-30 (kg/m^2^)	12	7.9
	Less than 25 kg/m^2^	139	92.1
Surgery type	Primary	130	86.1
	Secondary	21	13.9
Indication for surgery	Nodules or indurations	56	37.1
	Significant asymmetry	49	32.4
	Disfiguring facial edema	14	9.3
	Severe incapacitating deformities	32	21.2

Table 2 shows that the technique was highly successful, as failure to deliver the buccal pad of fat occurred in only 6.0% of surgeries. In addition, the procedure was associated with a low complication rate, including seromas (3.3%) and early infections (1.3%). Two patients had residual contour irregularities that needed touch up surgeries which were addressed using fat grafting. One patient was treated with two sessions of fat grafting with no complication whereas the second patient was managed by four sessions of fat grafting with three months spacing.

**Table 2:** Complications and patient satisfaction following combined buccal fat pad flap and deep plane facelift

Factors	Category	Number	Frequency
Surgery	Failed	9	6.0
	Done successfully	142	94.0
Previous infection	Yes	18	11.9
	No	133	88.1
Seromas	Yes	5	3.3
	No	146	96.7
Touch-up surgery	Yes	2	1.3
	No	149	98.7
Early infection	Yes	2	1.3
	No	149	98.7
Contour irregularity by the patient	Yes	5	3.3
	No	146	96.7
Contour irregularity by the surgeon	Yes	16	10.6
	No	135	89.4
Patient satisfaction	Excellent	57	37.7
	Very Good	65	43.0
	Fair	24	15.9
	Poor	5	3.3

Patient satisfaction was mainly high, with 80.7% reporting outcomes from Very Good to Excellent. Notably, surgeons identified contour irregularities at a rate of 10.6%, compared to just 3.3% reported by patients. Overall, this combined procedure is safe and effective, with strong patient acceptance.

**Fig. 1:**
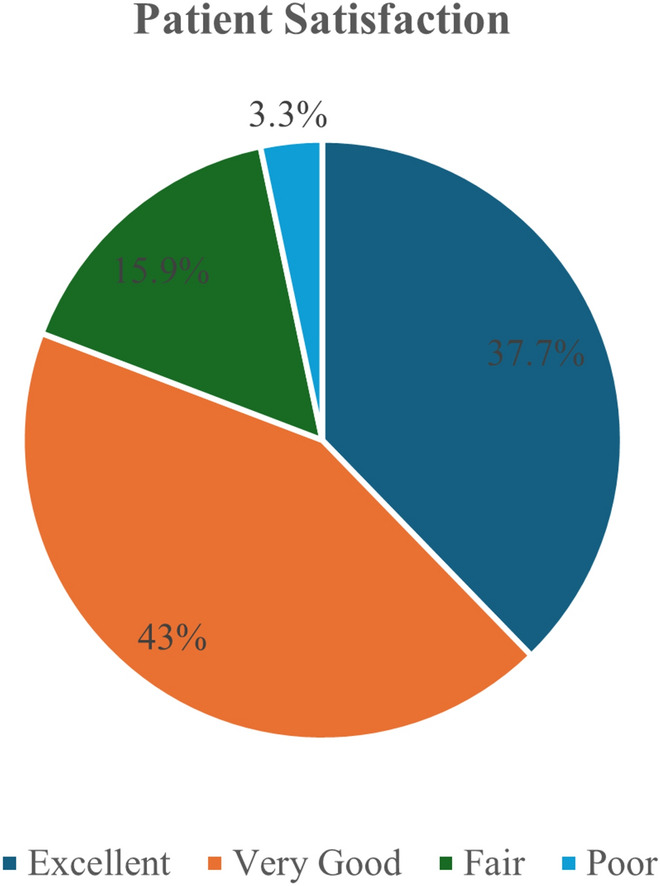
Patients satisfaction following the combined buccal pad of fat flap and deep plane facelift surgery

Figure 1 demonstrates high patient satisfaction with the combined procedure, as 80.7% of patients described their results to be “very good” or “excellent” (37.7% “excellent” and 43% “very good”).

Upon assessing the relation to preoperative variables, Table 3 indicates that patients who underwent primary surgery were significantly more satisfied than those who had secondary revisions. Specifically, 88.5% of the primary surgery group reported “Excellent” or “very good” results, in contrast to 33.4% in the secondary group (*p* < 0.001), reflecting increased technical challenges and tissue compromise in revision cases. Additionally, non-smokers exhibited higher satisfaction levels compared to smokers, with 85% reporting “Excellent” or “very good” results, compared to 64.5% among smokers (*p* = 0.011). Moreover, patients with a previous history of infection reported substantially lower levels of satisfaction (*p* = 0.006). However, the relation of variable indications, including nodules and indurations (*p* = 0.091), significant asymmetry (*p* = 0.464), disfiguring facial edema (*p* = 0.551), and severe incapacitating deformity (*p* = 0.439), was not statistically associated with patient satisfaction levels. Only the presence of a preoperative severe deformity showed a statistically significant association (*p* = 0.045) with the surgeon-noted contour irregularities.

**Table 3:** Patient satisfaction and the influence of surgical history, complications, and outcomes

Factors	Category	Patient satisfaction N (%)				*P*- Value
		Excellent	Very good	Fair	Poor	
Gender	Female	53 (37.9)	59 (42.1)	23 (16.4)	5 (3.6)	0.890
	Male	4 (36.4)	6 (54.5)	1 (9.1)	0 (0)	
Age group (years)	Less than or equal to 45	28 (38.4)	28 (38.4)	15 (20.5)	2 (2.7)	0.408
	More than 45	29 (37.2)	37 (47.4)	9 (11.5)	3 (3.8)	
Smoking status	Yes	9 (29.0)	11 (35.5)	7 (22.6)	4 (12.9)	**0.011**
	No	48 (40.0)	54 (45.0)	17 (14.2)	1 (0.8)	
BMI between 25-30 (kg/m^2^)	Yes	5 (41.7)	6 (50.0)	1 (8.3)	0 (0)	0.901
	No	52 (37.4)	59 (42.4)	23 (16.5)	5 (3.6)	
Surgery type	Primary	56 (43.1)	59 (45.4)	14 (10.8)	1 (0.8)	**<0.001**
	Secondary	1 (4.8)	6 (28.6)	10 (47.6)	4 (19.0)	
Nodules or indurations	Yes	15 (26.8)	31 (55.4)	8 (14.3)	2 (3.6)	0.091
	No	42 (44.2)	34 (35.8)	16 (16.8)	3 (3.2)	
Significant asymmetry	Yes	23 (46.9)	18 (36.7)	7 (14.3)	1 (2)	0.464
	No	34 (33.3)	47 (46.1)	17 (16.7)	4 (3.9)	
Disfiguring facial edema	Yes	5 (35.7)	5 (35.7)	3 (21.4)	1 (7.1)	0.551
	No	52 (38)	60 (43.8)	21 (15.3)	4 (2.9)	
Severe incapacitating deformities	Yes	15 (46.9)	10 (31.3)	6 (18.8)	1 (3.1)	0.439
	No	42 (35.3)	55 (46.2)	18 (15.1)	4 (3.4)	
Surgery	Failed	1 (11.1)	3 (33.3)	3 (33.3)	2 (22.2)	**0.010**
	Done successfully	56 (39.4)	62 (43.7)	21 (14.8)	3 (2.1)	
Previous infection	Yes	3 (16.7)	6 (33.3)	8 (44.4)	1 (5.6)	**0.006**
	No	54 (40.6)	59 (44.4)	16 (12.0)	4 (3.0)	
Seromas	Yes	0 (0)	2 (40.0)	2 (40.0)	1 (20.0)	0.024
	No	57 (39.0)	63 (43.2)	22 (15.1)	4 (2.7)	
Touch-up surgery	Yes	1 (50.0)	0 (0)	0 (0)	1 (50.0)	0.055
	No	56 (37.6)	65 (43.6)	24 (16.1)	4 (2.700)	
Early infection	Yes	0 (0)	2 (100)	0 (0)	0 (0)	0.670
	No	57 (38.3)	63 (42.3)	24 (16.1)	5 (3.4)	
Contour irregularity by the patient	Yes	0 (0)	1 (20.0)	4 (80.0)	0 (0)	0.007
	No	57 (39.0)	64 (43.8)	20 (13.7)	5 (3.4)	
Contour irregularity by the surgeon	Yes	1 (6.3)	5 (31.3)	10 (62.5)	0 (0)	**<0.001**
	No	56 (41.5)	60 (44.4)	14 (10.4)	5 (3.7)	

Significant *P* values are highlighted in bold

When it comes to complications, patients who experienced postoperative seroma had significantly lower satisfaction rates (*p* = 0.024), while there was no statistically significant association between surgical indications and the occurrence of complications such as surgical failure, seroma, early infection, or the need for secondary touch-up procedures. Moreover, the presence of specific indications, including nodules and irregularities (*p* = 0.091), asymmetry (*p* = 0.464), facial edema (*p* = 0.551), and severe deformity (*p* = 0.439), was not statistically associated with patient satisfaction levels.

The study highlights the correlation of surgeons’ evaluations and patient-reported outcomes, as contour irregularities reported by surgeons were strong predictors of dissatisfaction among patients. Particularly, 100% of patients who noted contour irregularities had asymmetries reported by surgeons, versus 7.5% of patients who were noted by the surgeons to have irregularities yet did not report concerns. On the other hand, other complications showed no significant association with demographic or clinical factors. Only the presence of a severe deformity showed a statistically significant association (*p* = 0.045) with the measured outcome, while nodules, asymmetry, and facial edema did not demonstrate significant correlations.

## Case Presentation

### Case 1

**Fig. 2:**
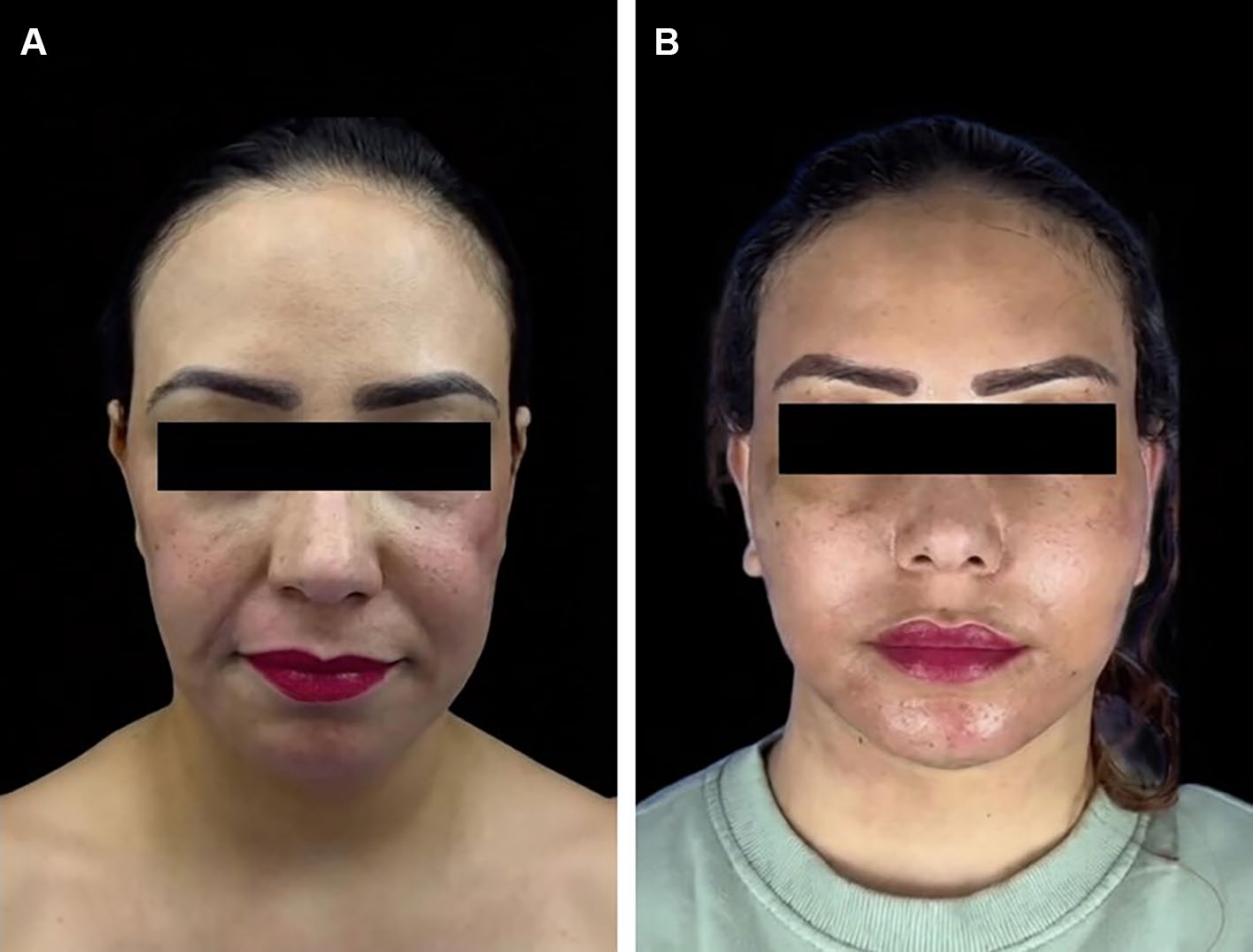
**A** Preoperative view of a 48-year-old female with previous permanent filler injection and significant deformity in her left cheek. **B** Postoperative results one year after treatment with the buccal pad of fad flap and deep plane facelift

A case of a 48-year-old woman with significant facial asymmetry and a depression in her left cheek. She had a history of permanent filler injection eight years prior. Her treatment involved removal of the permanent fillers, a deep plane facelift, and the use of a buccal fat pad flap to correct the defect in her left cheek. Figure 2 illustrates the results one year postoperatively.

### Case 2

**Fig. 3:**
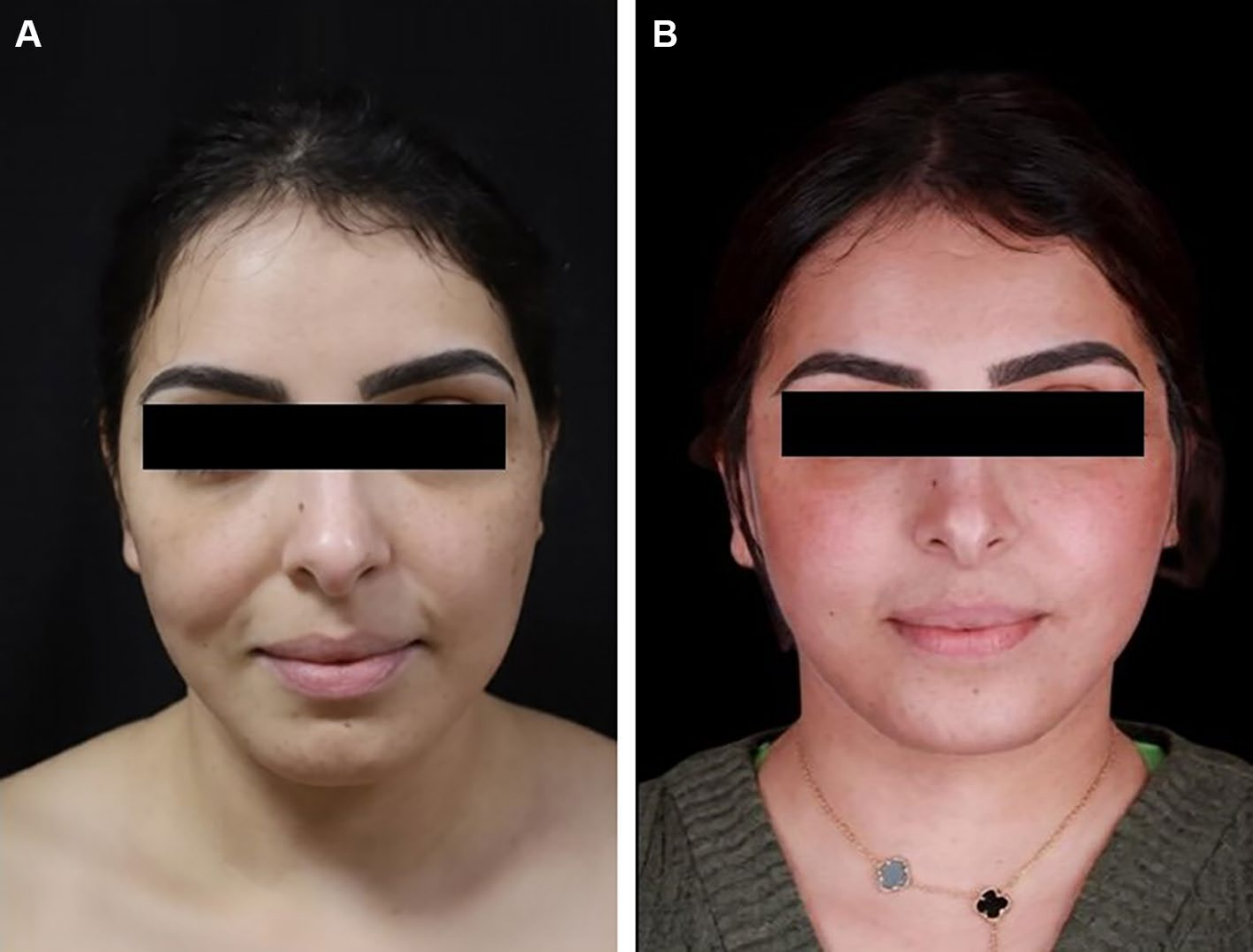
**A** Preoperative view of a 44-year-old female with previous permanent filler injection, removal of permanent fillers and using the buccal pad of fat flap to reconstruct mid face defect in both cheeks along with deep plane facelift was done. **B** Postoperative results after two years

Figure 3 shows the two-year-results after removal of permanent fillers and using the buccal pad of fat flap to reconstruct mid face defect in both cheeks. The 44-year-old patient gave a history of spontaneous acute infection on top of fillers in the right cheek 6 months before the surgery.

### Discussion

Permanent fillers is one of the most encountered challenges during facelift surgery. Extensive tissue scarring and defects detected within removal of the fillers mandate special reconstructive approaches [11, 25, 26]. After removing permanent fillers, additional filler injections are generally not advised due to limited evidence supporting their effectiveness as a reconstructive technique. The literature describes various reconstructive procedures to address such defects with significant focus on fat grafting [29–32]. Fat grafting has been a recognized method for correcting volume deficits and asymmetries post-permanent filler removal. A study involving 231 patients undergoing filler evacuation revealed that 22 of those patients required fat grafting, and half of those patients needed secondary procedures [13]. It is shown in literature that fat grafting offers a low risk of adverse reactions while restoring facial balance post-permanent filler removal [29, 31–33]. On the other hand, fat grafting combined with facelift procedures was associated with seromas at a rate of 8% and wound infections at a rate of 4% [34]. Facial fat grafting side effects are classified into three categories: severe (13.4%), moderate (38.3%), and minor (48.3%). Severe side effects, such as intravascular injection or migration, may require neurosurgery and could lead to permanent disability or death. Moderate side effects include fat hypertrophy, necrosis, and irregularities, often needing follow-up procedures. Minor side effects typically involve prolonged swelling or redness and usually do not require surgery. The overall complication rate is about 2% [35]. Another evidence on lipofilling following laser-assisted filler removal noted complications such as edema, bruising, infection, nodules, oily cysts, and persistent asymmetry, although specific rates were not provided [36].

The buccal fat offers a notable advantage due to its natural resistance to resorption and the straightforward harvesting process. Its use for treatment of facial soft tissue enhancement in conditions such as hemifacial microsomia, scar adhesion, and temporal hollowness has been descirbed [37]. Our research aimed to describe the outcomes of a comparative methodology to correct facial defects in patients who underwent permanent filler removal using a pedicled buccal pad of fat flap in conjunction with a deep plane facelift. The study shows that the pedicled buccal fat pad flap procedure achieved an 80.7% satisfaction rate with fewer complications in comparison to the widely adopted fat grafting procedure. This is shown by 3.3% of our candidates who experienced seroma, and early infection was detected in 1.3%. Only 1.3% required touch-up surgery, indicating greater durability. In addition, previous studies involving the pedicled flap reported minimal complications of the procedure, including a few incidences of hematoma, infection, facial nerve injury, partial necrosis, and scar formation [38–41]. Other identified complications following buccal fat flap surgery are flap necrosis, infections, and bleeding that can lead to hematoma formation [42–45].

We investigated the relationship between preoperative variables and postoperative complications with the success of the combined procedure. Our study did not find significant effects of gender on the occurrence of complications. However, the fact that males represented a small proportion (7.3% vs. 92.7% females) may affect the accuracy of our results regarding gender. Regarding age, the study found no significant difference on the occurrence of minor or major postoperative complications between the two age groups. This aligns with the research by Abboushi et al, who also found no significant association while observing their facelift procedures [46]. Similarly, a retrospective review of 216 facelift surgeries concluded the safety of the procedure in patients over 65 years old compared to those under 65 [47]. While some previous studies have identified gender as a risk factor [46, 48].

In this study, obese patients (BMI > 30) were excluded, which means we cannot determine how BMI affects the incidence of complications. However, Abboushi et al. found that complication rate among patients with a BMI higher than 25 undergoing facelift was 9.5%, compared to just 4.7% for normal-weight patients [46]. In addition, another multi-center study by Gupta et al. found that BMI is an independent predictor of complications in various cosmetic procedures [49]. Therefore, the increased complication rate as a result of higher BMI likely applies to our combined procedure as well.

In this study, the prevalence of smokers among patients was 20.5%. However, smoking status did not significantly affect the rate of complications following our procedure. Our results indicate that primary surgeries have significantly higher satisfaction rates (88.5%) than secondary cases (66.7%). This aligns with the understanding that revision surgeries require an understanding of anatomy, wound healing, and careful planning, as scar formation alters tissue characteristics and affects the surgical outcomes [50]. This highlights the importance of timely definitive interventions and the need for meticulous patient counseling regarding the limitations of revision surgery.

According to our findings, the most significant indicators linked to poor patient satisfaction following the buccal pad of fat flap surgery include smoking, history of previous procedures, and the development of post-operative complication specifically seroma. Additionally, we found a strong correlation between the absence of complications, particularly infections, and a higher rate of patient satisfaction.

To conclude, our findings align with previous reports describing the low complication rate associated with the buccal pad of fat flap. Still, we provide new insights by quantifying the most common issues: contour irregularities (10.6% as detected by surgeons and 3.3% as reported by patients) and seromas (9.2%). Additionally, the outcome of the buccal fat pad procedure has shown to be negatively affected by smoking, previous surgeries and the presence of post-operative complications.

The difference between the surgeon-reported and patient-reported contour irregularities highlights the importance of including patient-reported outcomes in future surgical evaluations.

### Clinical Implications

The buccal pad of fat flap surgery, combined with a deep-plane facelift, offers an important restorative solution for patients who need reconstruction after permanent filler removal. Additionally, the link between complications and contour irregularities underscores the need for effective strategies to anticipate and mitigate potential obstacles that may arise after these procedures.

## Limitations of the Study

This study is limited by its single-center design, and lack of a control group. Future investigations should focus on comparing different reconstructive techniques and examining the long-term outcomes in varied population groups. It is suggested that validated tools such as FACE-Q or SF-36 could be incorporated.

## Conclusion

Our technique of using the buccal fat pad flap in conjunction with a deep-plane facelift for patients requiring permanent filler extraction achieved high success rates and remarkable patient satisfaction, especially in primary procedures, where tissue integrity was maintained. Secondary revisions usually result in lower success and satisfaction rates due to the difficulties encountered. There was a strong correlation between patient satisfaction and the absence of complications, underscoring the importance of meticulous surgical technique and attentive postoperative management.

## Supplementary Information

Below is the link to the electronic supplementary material.

Supplementary file1 (MOV 13211 kb)
Buccal fat pad mobilization
